# Low Temperature Sealing of Anodized Aluminum Alloy for Enhancing Corrosion Resistance

**DOI:** 10.3390/ma13214904

**Published:** 2020-10-31

**Authors:** Hyunbin Jo, Soomin Lee, Donghyun Kim, Junghoon Lee

**Affiliations:** 1 Department of Metallurgical Engineering, Pukyong National University, 45, Yongso-ro, Nam-Gu, Busan 48513, Korea; jhbin@pukyong.ac.kr (H.J.); ktftnals@gmail.com (S.L.); 2 Analysis Technical Center, Korea Institute of Ceramic Engineering and Technology, 101, Soho-ro, Jinju-si, Gyeongsangnam-do 52851, Korea; dhkim1208@kicet.re.kr

**Keywords:** aluminum alloy, anodizing, sealing, corrosion

## Abstract

Sealing as a post treatment of anodized aluminum is required to enhance the corrosion resistance by filling nanopores, which allow the penetration of corrosive media toward the base aluminum. We designed a mixed sealing solution with nickel acetate and ammonium fluoride by modifying traditional nickel fluoride cold sealing. The concentration of mixed sealing solution affected the reaction rate of sealing and corrosion current density of anodized aluminum alloy. The higher concentration of mixed sealing solution improved the sealing rate, which was represented by a decrease of corrosion current density of anodized aluminum alloy. However, a mixed sealing solution with 2/3 concentration of general nickel fluoride sealing solution operated at room temperature showed the lowest corrosion current density compared to traditional methods (e.g., nickel fluoride cold sealing (NFCS) and nickel acetate hot sealing) and other mixed sealing solutions. Moreover, the mixed sealing solution with 2/3 concentration of general NFCS had a lower risk for over sealing, which increases the corrosion current density by excessive dissolution of anodic oxide. Therefore, the mixed sealing solution with optimized conditions designed in this work possibly provides a new method for enhancing the corrosion resistance of anodized aluminum alloys.

## 1. Introduction

Aluminum alloys are one of the most widely used metallic materials, from infrastructures to uses in our daily lives. There are various incentives for using aluminum alloys, such as a weight, thermal conductivity, machining, cold working, specific strength, castability and so on [1,2,3,4]. However, aluminum alloys have a serious corrosion issue in various application fields due to their relatively high chemical reactivity. In addition, aluminum alloys can be an anode by the galvanic contact with most of other commercial metals. Therefore, surface treatments creating a passivation layer, which inhibits the transportation of oxygen, humidity and corrosive media toward the base metal, are required for aluminum alloys [5,6,7].

Various strategies have been employed to impede the corrosion of aluminum alloys regarding the service environments. The most convenient way to form a passivation layer on aluminum is an organic coating, including painting, spraying and wrapping [8,9]. UV exposure and temperature change easily degrade the polymer layer to allow the transport of corrosive media, resulting in crevice corrosion and delamination of the coating layer [10,11,12]. Thermal spray coating a hard ceramic protective layer also reduces the mass transport toward metal substrates [13,14]. However, the porous nature of coating layers made of ceramic particles enables the transport of corrosive media [15,16]. Moreover, the thermal spray cannot create a uniform coating layer on complex shaped parts and some metals, which have a low melting temperature (e.g., aluminum). Electrodeposition/electroless deposition of other metals can be also employed to reduce the corrosion of aluminum alloys [17,18,19,20]. However, regarding the galvanic series, deposition of noble metals causes severe localized corrosion of aluminum alloy in damaged regions. Although a passive oxide layer is formed on aluminum alloys by chemical conversion coatings, the mechanical robustness of thin oxide layers and use of harmful chemicals still remain issues [21,22]. Sol–gel coatings forming a passive oxide layer have been employed for anti-corrosion of metals, but surface cracking and poor adhesion still remain unsolved issues [23,24,25]. Anodic oxidation (i.e., anodization or anodizing) creates a thick protective oxide layer by electrochemical reactions between ions and the aluminum substrate, which results in good adhesion of the oxide layer on the aluminum alloy substrate compared to thermal spray and sol–gel coatings [26,27,28]. Moreover, the porous nature of anodic oxide, which allows the transfer of corrosive media, can be densified by post treatments of anodization, such as a sealing [29,30].

Since the anodic aluminum oxide has nanoscale high-aspect-ratio cylindrical pores [31,32], which provide paths of corrosive media toward aluminum substrate causing the corrosion, various sealing methods to plug the nanopores with stable materials have been employed to improve the corrosion resistance of anodized aluminum alloys [31,32,33,34]. Due to the sealing treatment, the absorption and transportation of corrosive media in the nanopores of anodic aluminum oxide are significantly impeded, so that the corrosion resistance can be enhanced. Hot sealing methods require relatively short times for complete sealing of nanoporous anodic aluminum oxide, but the process consumes a huge amount of electric energy for boiling the water or aqueous solution [35,36,37,38,39]. Cold sealing methods operated at around room temperature have an advantage in processing cost [40]. However, cold sealing methods take a longer processing time for the complete sealing and use expensive chemicals (e.g., nickel fluoride) [36,41,42,43].

In this study, we designed a new aqueous solution for the sealing of nanoporous anodic aluminum oxide operating at around room temperature. Cold nickel fluoride sealing, which is one of the most well-known sealing methods, is modified by mixing fluorides and nickel based chemicals, so that the active ions are similar to the nickel fluoride. The anodic oxidation with sealing treatments were applied for aluminum alloy 6061, which is commonly used in marine and aerospace applications due to its superior mechanical properties. With respect to the concentration of mixed solution and processing time, the efficiency of new sealing methods is evaluated with a corrosion test using a potentiodynamic polarization, surface morphology and chemical composition analysis. Then the optimized conditions of mixed solution were explored for maximizing corrosion resistance of anodized aluminum alloy. The enhancements of corrosion resistance were also compared with traditional sealing methods, such as hot sealing in nickel acetate and cold sealing in nickel fluoride.

## 2. Materials and Methods

Aluminum alloy 6061-T6 (chemical composition: Fe: 0.24, Si: 0.15, Al: balance (wt.%); thickness: 1 mm) cut into 3 cm × 5 cm was used as a substrate. The sample was degreased in acetone and a detergent solution for 3 min with ultrasonication, then rinsed with deionized water. The surface of the aluminum sample was electropolished in a perchloric acid and ethanol mixture (1:4 in volumetric ratio) under constant voltage of 20 V for 7 min at 5 °C. The aluminum alloy substrate was anodized under constant voltage of 17 V in 2.1 M H_2_SO_4_ solution at 21 °C for 30 min.

The anodized aluminum sample was sealed in various solutions and duration, as shown in Table 1. Considering the reaction rate in each sealing solution, the immersion time was varied. Then, the sample was cleaned in deionized water, and the residual water was blown away with compressed air. Nickel acetate hot sealing and nickel fluoride cold sealing are well-known processes used in the practical manufacturing industry. New sealing solutions are mixtures of nickel acetate and ammonium fluoride with various concentrations.

Corrosion resistance of anodized aluminum alloy with various sealing treatments was evaluated by a potentiodynamic polarization test in 3.5 wt.% NaCl solution at room temperature using a potentiostat (VersaSTAT 4, Princeton Applied Research, Oak Ridge, TN, USA). A three electrode flat cell with platinum mesh (counter electrode) and saturated calomel electrode (SCE, reference electrode) was used. Prior to the potential scan, the sample was immersed in the electrolyte for 30 min to stabilize the open circuit potential (OCP). Then the potential was scanned −400 mV to 800 mV vs. OCP with 2 mV/s rate. Seven samples freshly fabricated with the same conditions were used for a potentiodynamic polarization test. Then, the corrosion current density of each sample was estimated with Tafel fitting and averaged without maximum and minimum value [44]. The surface chemical composition and morphology of the anodized aluminum with various sealings were analyzed by using energy dispersive spectroscopy (EDS) and field-emission scanning electron microscope (FE-SEM).

## 3. Results and Discussion

**Figure 1:**
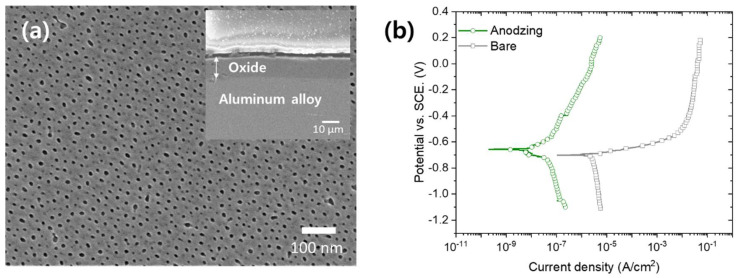
(**a**) SEM images and (**b**) potentiodynamic polarization curve (in 3.5 wt.% NaCl solution) of anodized aluminum alloy 6061.

The anodizing of aluminum alloy 6061 substrate under 17 V for 30 min resulted in a nanoporous oxide layer with a pore diameter of 15 nm and thickness of 11 μm (Figure 1a). Figure 1b shows potentiodynamic polarization curves of bare and anodized aluminum alloy 6061 measured in 3.5 wt.% NaCl solution. This nanoporous oxide layer protected the aluminum metal substrate from the corrosive environment by inhibiting the transfer reactive media; thus, the corrosion current density of anodized aluminum alloy decreased from 2.59 × 10^−6^ (bare surface) A/cm^2^ to 3.45 × 10^−8^ A/cm^2^ with an inhibition efficiency of 98.67%. The corrosion current density of anodized aluminum alloy can be further significantly decreased by a sealing treatment, which fills nanopores of oxide layer, so that a penetration of corrosive media toward base aluminum is inhibited [5].

**Figure 2:**
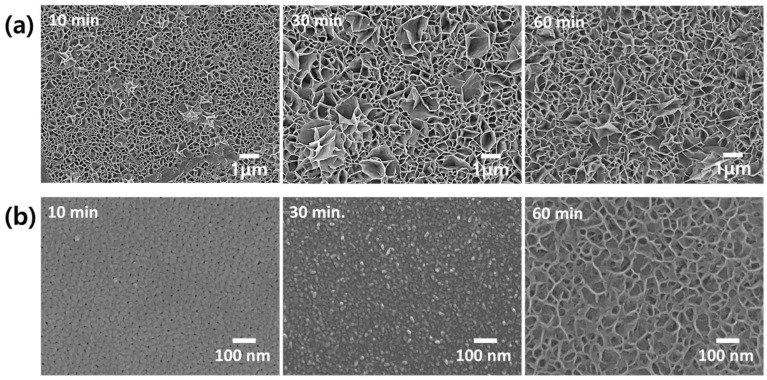
Surface SEM images of nanoporous anodic aluminum oxide with (**a**) nickel acetate hot sealing (NAHS) and (**b**) nickel fluoride cold sealing (NFCS) for 10 min, 30 min and 60 min.

**Figure 3:**
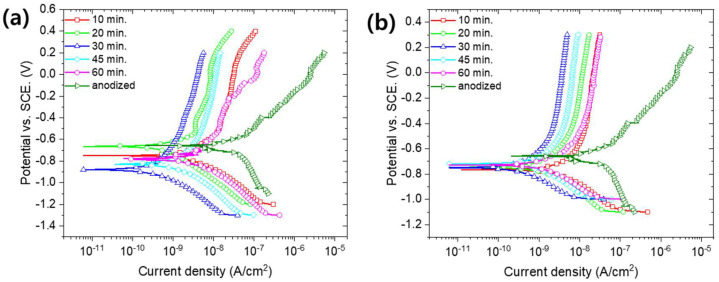
Potentiodynamic polarization curves of anodized aluminum alloy 6061 with (**a**) nickel acetate hot sealing (NAHS) and (**b**) nickel fluoride cold sealing (NFCS).

**Figure 4:**
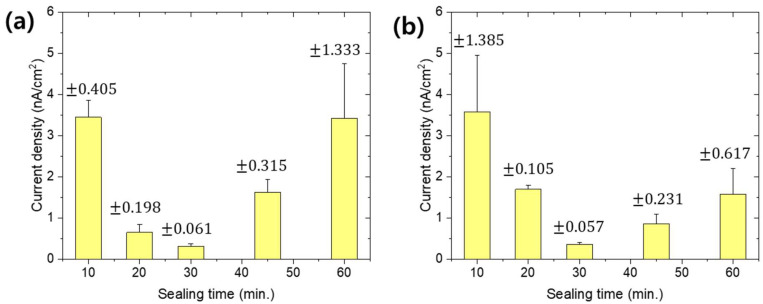
Estimated corrosion current density from potentiodynamic polarization curves (Figure 3) for (**a**) NAHS and (**b**) NFCS with respect to various sealing times.

Nickel acetate hot sealing (NAHS) and nickel fluoride cold sealing (NFCS) are the most well-known treatments for anodized aluminum alloy to enhance the corrosion resistance. Figure 2 shows the SEM surface image of nanoporous anodic aluminum oxide with NAHS and NFCS as a function of treatment time. Figure 3 shows the potentiodynamic polarization curves of anodized aluminum alloy with NAHS and NFCS. In addition, the corrosion current density of each curve was estimated and then summarized in Figure 4. A nanoscale cellular structure was formed on the nanoporous anodic aluminum oxide by the NAHS for 10 min, and then the cellular structure was enlarged with the increase of processing time (Figure 2a). The concentrations of oxygen and nickel (Table 2) on the oxide surface were increased by NAHS, indicating that the nanoscale cellular structure was composed of a boehmite (AlOOH) and nickel hydroxide (Ni(OH)_2_) [35,36]. In addition, the formation reaction of boehmite and nickel hydroxide occurred in the nanopores, so that the penetration of corrosive media through the nanopores could be suppressed [36]. As a result of boehmite and nickel hydroxide formation on the surface and in the pores, the corrosion current density further decreased from 3.45 × 10^−8^ (as anodized) A/cm^2^ to 3.45 × 10^−9^ A/cm^2^, to 0.65 × 10^−9^ A/cm^2^, to 0.31 × 10^−9^ A/cm^2^, to 1.62 × 10^−9^ A/cm^2^ and to 3.42 × 10^−9^ A/cm^2^ for 10 min, 20 min, 30 min, 45 min and 60 min of NAHS, respectively. The corrosion current density of anodized aluminum alloy with NAHS decreased with the treatment time up to 30 min, and then it increased with the treatment time. Therefore, the efficacy of NAHS for 30 min was the maximum to enhance the corrosion resistance of nanoporous anodic oxide with an inhibition efficiency of 99.11%.

NFCS also filled the nanopores of anodized aluminum alloy at around room temperature, so that the diameter of pores shrunk with the treatment time. Therefore, smaller pores on the oxide surface with 10 min of NFCS than the anodized one could be observed (Figure 2b). Then, the NFCS of nanoporous anodic aluminum oxide for 30 min resulted in complete sealing, so that the pore could not be observed on the surface. Due to the NFCS, the increased chemical composition of oxygen and nickel on the surface (Table 2) indicated that the aluminum hydroxide (Al(OH)_3_) and nickel hydroxide (Ni(OH)_2_) were formed [36]. Instead of boehmite (AlOOH), which is formed by NAHS at temperatures higher than 80 °C, NFCS created aluminum hydroxide (Al(OH)_3_). In addition, an increase of fluorine indicated the formation of aluminum fluoride (AlF_3_) [41]. These hydroxides and fluoride filled the nanopores of anodic oxide; thus, the corrosion current density of anodized aluminum alloy with respect to the NFCS time decreased from 3.45 × 10^−8^ A/cm^2^ (as anodized) to 3.58 × 10^−9^ A/cm^2^, to 1.69 × 10^−9^ A/cm^2^ and to 0.35 × 10^−9^ A/cm^2^ for 10, 20 and 30 min of sealing time, respectively. However, the surface of anodic oxide with 60 min of NFCS showed a nanoscale cellular structure (Figure 2b), which was due to the overreaction dissolving the anodic oxide. Therefore, the corrosion current density of anodized aluminum alloy increased to 0.85 × 10^−9^ A/cm^2^ and to 1.58 × 10^−9^ A/cm^2^ for 45 min and 60 min of NFCS, respectively. These results show that 30 min of NFCS was the optimized condition to reduce the corrosion current density of anodized aluminum alloy with the inhibition efficiency of 98.99%.

Even though the NAHS and NFCS improved the corrosion resistance of aluminum alloy, the corrosion current density was not continually decreased with the sealing time. Therefore, it was important to find the optimized sealing time for minimizing the corrosion current density. In this study, 30 min of NAHS and NFCS was the optimized time to seal the anodized aluminum alloy, and they showed similar corrosion current densities. However, since the corrosion current density of anodized aluminum alloy with NAHS for more than 30 min increased more rapidly (compare Figure 4a,b) than NFCS, a stricter time control of NAHS was required for maximizing the corrosion resistance of anodized aluminum alloy. In addition, the NFCS only required 25 °C, while the NAHS was conducted at 95 °C. Therefore, the NFCS also has economic and environmental incentives compared to NAHS.

**Figure 5:**
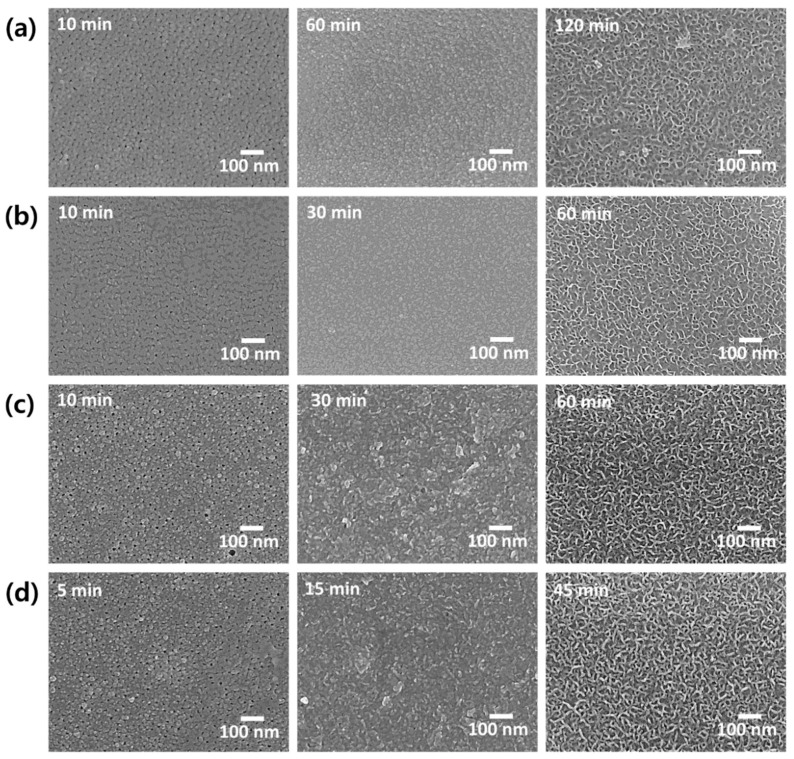
Surface SEM images of anodized aluminum alloy with sealing in mixed solution (**a**) MSS1, (**b**) MSS2, (**c**) MSS3 and (**d**) MSS4 with respect to various treatment times.

In this study, a new sealing solution for anodized aluminum alloy was designed by modifying the NFCS, which uses Ni^2+^ and F^−^ ions for the sealing reaction. We mixed the nickel acetate and ammonium fluoride as a source of Ni^2+^ and F^−^ ions, respectively. Ion concentrations of the designed sealing solution (nickel acetate + ammonium fluoride) MSS1, MSS1, MSS3 and MSS4 are equivalent to 1/3, 2/3, 1 and 2 of ion concentration of NFCS, respectively. Figure 5 shows surface SEM images of anodized aluminum alloy with sealing in MSS1, MSS2, MSS3 and MSS4 for various durations. The nanopores of anodic oxides were not fully filled in after 10 min using MSS1, MSS2, MSS3 and MSS4, and thus the nanopores were still observable. It took 60 min to fully fill the nanopores of anodic oxide using MSS1, which had 1/3 the ion concentration of NFCS. In the case of MSS2 and MSS3 containing 2/3 and equivalent ion concentration of NFCS, the surface pores were fully sealed in 30 min, and then the anodic oxides sealed in MSS2 and MSS3 for 60 min showed the nanocellular structure. The pores were fully sealed in 15 min using MSS4, which had a higher ion concentration than NFCS. Then, the surface of anodic oxide was overreacted to form the nanocellular structure in 30 min using MSS4. These results indicate that the concentration of Ni^2+^ and F^−^ affects the sealing reaction rate, and thus the higher concentration of sealing solution shortens the treatment time for complete sealing of nanoporous anodic oxide.

The ion concentration of MSS1-4 also affected the chemical composition of sealed nanoporous anodic oxide (Table 3). The sealing of anodic aluminum oxide in mixed solution with nickel acetate and ammonium fluoride caused an increase of nickel and fluorine concentration on the surface. The dissolution of anodic aluminum oxide in the sealing solution caused an increase of pH at the oxide/solution interface, which enabled precipitation of nickel hydroxide on the oxide surface. Therefore, the nickel detected on the surface indicated the formation of nickel hydroxide (Ni(OH)_2_), and the concentration of nickel increased with the concentration of mixed sealing solution, affecting the dissolution of anodic aluminum oxide [41]. The fact that the time for complete sealing was decreased with the concentration of sealing solution also suggests the faster dissolution rate of aluminum oxide formed from the anodizing. Therefore, even though the nickel hydroxide precipitated on the oxide surface, the sealed nanoporous anodic oxides showed different anti-corrosion performances with respect to the concentration of sealing solutions.

**Figure 6:**
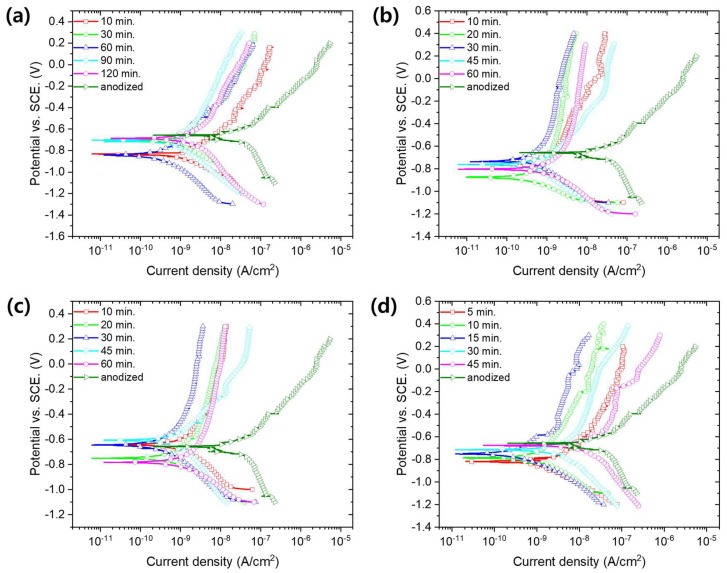
Potentiodynamic polarization curves of anodized aluminum alloy 6061 with sealing in mixed solution (**a**) MSS1, (**b**) MSS2, (**c**) MSS3 and (**d**) MSS4 with respect to various treatment times.

**Figure 7:**
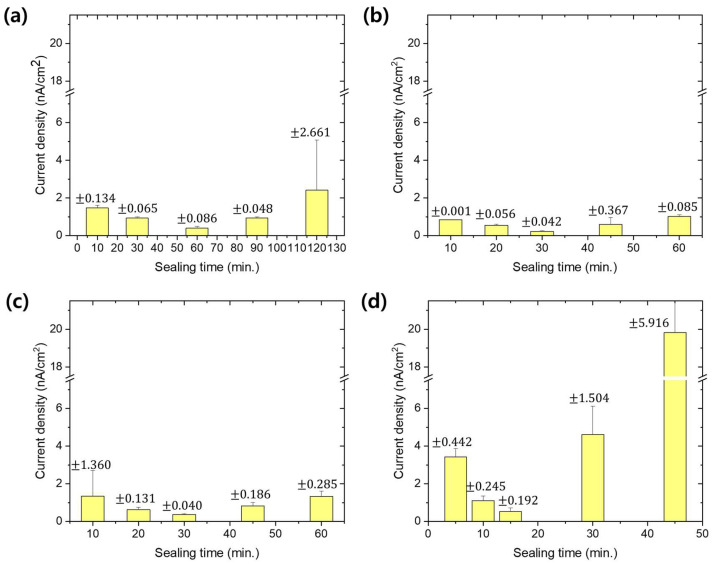
Estimated corrosion current density from potentiodynamic polarization curves (Figure 5) for anodized aluminum alloy with sealing in mixed solution (**a**) MSS1, (**b**) MSS2, (**c**) MSS3 and (**d**) MSS4 with respect to various treatment times.

Figure 6 shows the potentiodynamic polarization curves of anodized aluminum alloy with sealing in MSS1-4 solutions. Averaged corrosion current density of each anodized sample with sealing is also shown in Figure 7 as a function of treatment time. The sealing of anodized aluminum alloy with solution MSS1 containing 1/3 Ni^2+^ and F^−^ concentration of NFCS showed a decrease of corrosion current density up to 60 min of sealing time, such as 1.47 × 10^−9^ A/cm^2^ for 10 min, 0.93 × 10^−9^ A/cm^2^ for 30 min and 0.41 × 10^−9^ A/cm^2^ for 60 min. Then, the corrosion current density of anodized aluminum alloy was gradually increased with the sealing time in MSS1 for more than 60 min, such as 0.94 × 10^−9^ A/cm^2^ for 90 min and 2.41 × 10^−9^ A/cm^2^ for 120 min. Since it took 60 min to completely seal the nanopores of anodic oxide in MSS1 (Figure 5a), the corrosion current density of anodized aluminum alloy with sealing in MSS1 for 60 min was also the minimum. Nevertheless, the corrosion current density of the sample with sealing in MSS1 for 60 min was greater than the cases of NFCS and NAHS for 30 min by more than two-fold. The low concentration (Ni^2+^ and F^−^) of mixed solution MSS1 showed not only a low reaction rate for sealing but also an insufficient efficiency for enhancing the corrosion resistance of anodized aluminum alloy.

In the case of sealing with MSS2, including 2/3 of the ion concentration of NFCS, the corrosion current density of anodized aluminum alloy dramatically decreased to 0.84 × 10^−9^ A/cm^2^ in 10 min of sealing time, which was a much smaller corrosion current density than the cases of NFCS and NAHS for 10 min. Since the nanopores of anodic oxide were completely sealed in MSS2 for 30 min (Figure 5b), the corrosion current density gradually decreased to 0.54 × 10^−9^ A/cm^2^ for 20 min and to 0.22 × 10^−9^ A/cm^2^ for 30 min. Then, due to the excessive dissolution of anodic aluminum oxide in MSS2 after 30 min, the corrosion current density increased with the sealing time for more than 30 min, such as 0.59 × 10^−9^ A/cm^2^ for 45 min, and 1.02 × 10^−9^ A/cm^2^ for 60 min. Moreover, even though MSS2 included less Ni^2+^ ion concentration than traditional solutions (NAHS and NFCS), the corrosion current density of anodized aluminum alloy sealed in MSS2 for 30 min was 71% of NAHS and 62% of NFCS. These results indicate that the sealing in MSS2 not only showed a better efficiency in 30 min of sealing duration but also showed higher corrosion resistance of anodized aluminum alloy than the traditional methods (NAHS and NFCS). Moreover, the sealing in MSS2 had a slower increase rate of corrosion current density with a sealing time of more than 30 min compared to traditional methods, indicating that the sealing in MSS2 had a lower risk of over-sealing. 

In the case of MSS3, which had equivalent Ni^2+^ and F^–^ ion concentration with NFCS, the corrosion current density of anodized aluminum decreased to 1.34 × 10^−9^ A/cm^2^, 0.62 × 10^−9^ A/cm^2^, 0.36 × 10^−9^ A/cm^2^, 0.82 × 10^−9^ A/cm^2^ and 1.32 × 10^−9^ A/cm^2^ for 10 min, 20 min, 30 min, 45 min and 60 min of sealing time, respectively. Similar to MSS2, since the pores of anodic oxide were almost completely filled in 30 min (Figure 5c), the corrosion current density decreased up to 30 min of sealing time. Then, due to the over-sealing transforming the anodic oxide to become more porous, the corrosion current density increased with the sealing time for more than 30 min. Even though the ion concentration of MSS3 was greater than MSS2, the sealing rate using MSS3 was similar with MSS2. However, the anodized aluminum alloy with sealing in MSS3 showed higher corrosion current densities than the sealing in MSS2 for each sealing duration. In addition, the efficiency of MSS3 to reduce the corrosion current density of anodized aluminum was comparable to the traditional methods (NAHS and NFCS).

Higher ion concentration (Ni^2+^ and F^−^) of MSS4 than the traditional methods (NAHS and NFCS) shortened the time for complete filling of nanopores (Figure 5d). Therefore, the corrosion current density of anodized aluminum alloy rapidly decreased with respect to the sealing time in MSS4 up to 15 min. Then, due to the overreaction dissolving the anodic oxide, the corrosion current density increased with the sealing time in MSS4 for more than 15 min. Even though the MSS4 showed a fast reaction rate for sealing, the corrosion current density was higher than the cases of MSS1, MSS2 and MSS3 in each sealing duration. Moreover, the minimum corrosion current density of anodized aluminum alloy sealed in MSS4 was much higher than the sample prepared with the traditional methods (NAHS and NFCS). Therefore, the mixed sealing solution MSS4 was ineffective to enhance the corrosion resistance of anodized aluminum alloy compared to the traditional methods.

## 4. Conclusions

A mixed sealing solution with nickel acetate and ammonium fluoride modified by nickel fluoride cold sealing showed efficacy to enhance the corrosion resistance of anodized aluminum alloy. With respect to the sealing time, the corrosion current density was decreased by filling nanopores of anodic aluminum oxide. Then, due to the excessive dissolution of anodic oxide in the sealing solution, the corrosion current density gradually increased with sealing duration. Therefore, each sealing method of anodized aluminum alloy had an optimized time, which showed minimum corrosion current density with respect to the sealing duration. The increase of ion concentration of the mixed sealing solution shortened the optimized sealing duration. However, the anodized aluminum alloy sealed in mixed solution containing 0.017 M nickel acetate and 0.035 M ammonium fluoride for 30 min showed not only the lowest corrosion current density among the cases for mixed sealing solutions but also better efficiency than the traditional sealing methods, such as nickel acetate hot sealing and nickel fluoride cold sealing. Therefore, the mixed sealing solution with nickel acetate and ammonium fluoride can be a promising candidate for post treatment of anodized aluminum alloy to enhance the corrosion resistance.

## Figures and Tables

**Table 1:** Experimental conditions for the sealing of anodic aluminum oxide.

Name	Composition	Ni^2+^ (g/L)	Immersion Time (min)	Temperature (°C)
NAHS ^1^	Ni(CH_3_CO_2_)_2_∙4H_2_O: 5.5 g/L (0.221 M) H_3_BO_3_: 8.2 g/L (0.133 M)	1.3	10, 20, 30, 45, 60	95
NFCS ^2^	NiF_2_∙4H_2_O: 4.35 g/L (0.026 M)	1.5	10, 20, 30, 45, 60	25
MSS1 ^3^	Ni(CH_3_CO_2_)_2_∙4H_2_O: 2.14 g/L (0.009 M) NH_4_F: 0.64 g/L (0.017 M)	0.5	10, 30, 60, 90, 120	25
MSS2 ^3^	Ni(CH_3_CO_2_)2∙4H_2_O: 4.28 g/L (0.017 M) NH_4_F: 1.28 g/L (0.035 M)	1.0	10, 20, 30, 45, 60	25
MSS3 ^3^	Ni(CH_3_CO_2_)_2_∙4H_2_O: 6.42 g/L (0.026 M) NH_4_F: 1.92 g/L (0.052 M)	1.5	10, 20, 30, 45, 60	25
MSS4 ^3^	Ni(CH_3_CO_2_)_2_∙4H_2_O: 12.84 g/L (0.052 M) NH_4_F: 3.84 g/L (0.104 M)	3.0	5, 10, 15, 30, 45	25

^1^ Nickel acetate hot sealing. ^2^ Nickel fluoride cold sealing. ^3^ Mixed sealing solution.

**Table 2:** Chemical composition (in at.%) of anodized aluminum alloy with nickel acetate hot sealing (NAHS) and nickel fluoride cold sealing (NFCS) analyzed by EDS.

Name	Al	O	Ni	F
As Anodized	36.6 ± 1.4	63.2 ± 3.0	0.22 ± 0.1	0.1 ± 0.1
NAHS	26.4 ± 1.9	71.0 ± 2.3	2.63 ± 1.1	–
NFCS	22.3 ± 1.7	69.0 ± 2.1	1.53 ± 0.8	7.2 ± 1.2

**Table 3:** Chemical composition (in at.%) of anodized aluminum alloy completely sealed in mixed solution analyzed by EDS.

Name	Al	O	Ni	F
MSS1	28.1 ± 3.5	61.3 ± 3.7	2.2 ± 0.7	8.4 ± 1.2
MSS2	25.9 ± 2.2	62.8 ± 4.0	3.3 ± 1.1	8.0 ± 1.3
MSS3	23.7 ± 3.0	64.3 ± 3.3	5.0 ± 1.9	7.1 ± 1.8
MSS4	21.4 ± 2.7	66.5 ± 4.5	6.0 ± 1.5	6.2 ± 2.0

## References

[1] Shih H.-H., Tzou S.-L. (2000). Study of anodic oxidation of aluminum in mixed acid using a pulsed current. Surf. Coat. Technol..

[2] González J., Morcillo M., Escudero E., López V., Otero E. (2002). Atmospheric corrosion of bare and anodized aluminium in a wide range of environmental conditions. Part I: Visual observations and gravimetric results. Surf. Coat. Technol..

[3] López V., González J., Otero E., Escudero E., Morcillo M. (2002). Atmospheric corrosion of bare and anodised aluminium in a wide range of environmental conditions. Part II: Electrochemical responses. Surf. Coat. Technol..

[4] Aerts T., Dimogerontakis T., De Graeve I., Fransaer J., Terryn H. (2007). Influence of the anodizing temperature on the porosity and the mechanical properties of the porous anodic oxide film. Surf. Coat. Technol..

[5] Wang F., Liu J., Li Y., Wang Y. (2011). Novel composite nanofilm of electropolymerization and self-assembling on AA5052 surface as anticorrosion coating. J. Appl. Polym. Sci..

[6] Hakimizad A., Raeissi K., Ashrafizadeh F. (2012). A comparative study of corrosion performance of sealed anodized layers of conventionally colored and interference-colored aluminium. Surf. Coat. Technol..

[7] Moutarlier V., Gigandet M., Normand B., Pagetti J. (2005). EIS characterisation of anodic films formed on 2024 aluminium alloy, in sulphuric acid containing molybdate or permanganate species. Corros. Sci..

[8] Leth-Olsen H., Nisancioglu K. (1997). Filiform Corrosion Morphologies on Painted Aluminum Alloy 3105 Coil Material. Corrosion.

[9] Hu R.-G., Zhang S., Bu J.-F., Lin C.-J., Song G.-L. (2012). Recent progress in corrosion protection of magnesium alloys by organic coatings. Prog. Org. Coat..

[10] Pathak S., Khanna A. (2009). Investigation of anti-corrosion behavior of waterborne organosilane–polyester coatings for AA6011 aluminum alloy. Prog. Org. Coat..

[11] Dong C., Sheng H., An Y., Li X., Xiao K., Cheng Y. (2010). Corrosion of 7A04 aluminum alloy under defected epoxy coating studied by localized electrochemical impedance spectroscopy. Prog. Org. Coat..

[12] Ni L., Chemtob A., Croutxé-Barghorn C., Moreau N., Bouder T., Chanfreau S., Pébère N. (2014). Direct-to-metal UV-cured hybrid coating for the corrosion protection of aircraft aluminium alloy. Corros. Sci..

[13] Leivo E., Vippola M.S., Sorsa P.P.A., Vuoristo P., Mäntyla T.A. (1997). Wear and corrosion properties of plasma sprayed Al_2_O_3_ and Cr_2_O_3_ coatings sealed by aluminum phosphates. J. Therm. Spray Technol..

[14] Knuuttila J., Sorsa P., Mäntylä T. (1999). Sealing of thermal spray coatings by impregnation. J. Therm. Spray Technol..

[15] Uozato S., Nakata K., Ushio M. (2003). Corrosion and wear behaviors of ferrous powder thermal spray coatings on aluminum alloy. Surf. Coat. Technol..

[16] Magnani M., Suegama P., Espallargas N., Dosta S., Fugivara C., Guilemany J., Benedetti A. (2008). Influence of HVOF parameters on the corrosion and wear resistance of WC-Co coatings sprayed on AA7050 T7. Surf. Coat. Technol..

[17] Hu J.-M., Liu L., Zhang J.-Q., Cao C.-N. (2007). Electrodeposition of silane films on aluminum alloys for corrosion protection. Prog. Org. Coat..

[18] Ghanbari S., Mahboubi F. (2011). Corrosion resistance of electrodeposited Ni–Al composite coatings on the aluminum substrate. Mater. Des..

[19] Hu J.-M., Liu L., Zhang J.-Q., Cao C.-N. (2006). Effects of electrodeposition potential on the corrosion properties of bis-1,2-[triethoxysilyl] ethane films on aluminum alloy. Electrochimica Acta.

[20] Wu L.-K., Liu L., Li J., Hu J.-M., Zhang J.-Q., Cao C.-N. (2010). Electrodeposition of cerium (III)-modified bis-[triethoxysilypropyl]tetra-sulphide films on AA2024-T3 (aluminum alloy) for corrosion protection. Surf. Coat. Technol..

[21] Xingwen Y., Chunan C., Zhiming Y., Derui Z., Zhongda Y. (2001). Study of double layer rare earth metal conversion coating on aluminum alloy LY12. Corros. Sci..

[22] Yang X., Tallman D., Gelling V., Bierwagen G., Kasten L., Berg J. (2001). Use of a sol–gel conversion coating for aluminum corrosion protection. Surf. Coat. Technol..

[23] Zheludkevich M., Serra R., Montemor M., Yasakau K.A., Salvado I.M.M., Ferreira M.G.S. (2005). Nanostructured sol–gel coatings doped with cerium nitrate as pre-treatments for AA2024-T3. Electrochimica Acta.

[24] Schem M., Schmidt T.F., Gerwann J., Wittmar M., Veith M., Thompson G., Molchan I., Hashimoto T., Skeldon P., Phani A. (2009). CeO_2_-filled sol–gel coatings for corrosion protection of AA2024-T3 aluminium alloy. Corros. Sci..

[25] Whelan M., Cassidy J., Duffy B. (2013). Sol–gel sealing characteristics for corrosion resistance of anodised aluminium. Surf. Coat. Technol..

[26] Diggle J.W., Downie T.C., Goulding C.W. (1969). A Study of the Formation and Dissolution of Porous Anodic Oxide Films on Aluminum: Behavior of the Porous Layer. J. Electrochem. Soc..

[27] Li F., Zhang L., Metzger R.M. (1998). On the Growth of Highly Ordered Pores in Anodized Aluminum Oxide. Chem. Mater..

[28] Bai A., Hu C.-C., Yang Y.-F., Lin C.-C. (2008). Pore diameter control of anodic aluminum oxide with ordered array of nanopores. Electrochimica Acta.

[29] Hoar T., Wood G. (1962). The sealing of porous anodic oxide films on aluminium. Electrochimica Acta.

[30] Mansfeld F., Chen C., Breslin C.B., Dull D. (1998). Sealing of Anodized Aluminum Alloys with Rare Earth Metal Salt Solutions. J. Electrochem. Soc..

[31] Lee W., Ji R., Gösele U., Nielsch K. (2006). Fast fabrication of long-range ordered porous alumina membranes by hard anodization. Nat. Mater..

[32] Stępniowski W.J., Bojar Z. (2011). Synthesis of anodic aluminum oxide (AAO) at relatively high temperatures. Study of the influence of anodization conditions on the alumina structural features. Surf. Coat. Technol..

[33] Lee J., Jung U., Kim W., Chung W. (2013). Effects of residual water in the pores of aluminum anodic oxide layers prior to sealing on corrosion resistance. Appl. Surf. Sci..

[34] Wu Y., Zhao W., Yu J., Xue Q. (2018). Influence of the Self-Sealing Layer on the Corrosion of Anodic Aluminum Oxide Films. ACS Appl. Nano Mater..

[35] Gonzalez J., Lopez V., Otero E., Bautista A., Lizarbe R., Barba C., Baldonedo J. (1997). Overaging of sealed and unsealed aluminium oxide films. Corros. Sci..

[36] Hao L., Cheng B.R. (2000). Sealing processes of anodic coatings—Past, present, and future. Met. Finish..

[37] Hu N., Dong X., He X., Browning J.F., Schaefer D.W. (2015). Effect of sealing on the morphology of anodized aluminum oxide. Corros. Sci..

[38] García-Alonso M.C., Escudero M.L., González-Carrasco J.L. (2001). Corrosion, Evaluation of the scale integrity in the Al2O3/MA956 system at different polarisation by using EIS. Mater. Corros..

[39] Lopez V., Gonzalez J., Bautista A., Otero E., Lizarbe R., Bautista A. (1998). The response of anodized materials sealed in acetate-containing baths to atmospheric exposure. Corros. Sci..

[40] Otero E., Lopez V., Gonzalez J.A. (1996). Aging of Cold-Sealed Aluminum Oxide Films at Room Temperature and at 50 C. Plat. Surf. Finish.

[41] Kalantary M.R., Gabe D.R., Ross D.H. (1992). A model for the mechanism of nickel fluoride cold sealing of anodized aluminium. J. Appl. Electrochem..

[42] Kalantary M.R., Gabe D.R., Ross D.H. (1993). Sealing of electrolytically formed porous films of aluminum by nickel fluoride process. Methods.

[43] Zuo Y., Zhao P.-H., Zhao J.-M. (2003). The influences of sealing methods on corrosion behavior of anodized aluminum alloys in NaCl solutions. Surf. Coat. Technol..

[44] Mansfeld F. (1973). Tafel Slopes and Corrosion Rates from Polarization Resistance Measurements. Corrosion.

